# Conditional Inactivation of *Nf1* and *Pten* in Schwann Cells Results in Abnormal Neuromuscular Junction Maturation

**DOI:** 10.1534/g3.118.200795

**Published:** 2018-11-27

**Authors:** Xiao-Xiao Li, Shi-Jie Zhang, Amy P. Chiu, Lilian H. Lo, Jeffery C. To, He-Ning Cui, Dewi K. Rowlands, Vincent W. Keng

**Affiliations:** * Department of Applied Biology and Chemical Technology, The Hong Kong Polytechnic University, Kowloon, Hong Kong SAR; † Department of Neurology, The First Affiliated Hospital, Guangzhou Medical University, Guangzhou, China; ‡ Institute of Clinical Pharmacology, Guangzhou University of Chinese Medicine, Guangzhou, China; § Laboratory Animal Services Centre, The Chinese University of Hong Kong, Sha Tin, New Territories, Hong Kong SAR

**Keywords:** neuromuscular junction, Schwann cell, Neurofibromin 1, phosphatase and tensin homolog

## Abstract

The neuromuscular junction (NMJ) consists of three components, namely presynaptic motor neurons, postsynaptic muscle fibers and perisynaptic Schwann cells (PSCs). The role of Schwann cells (SCs) in regulating NMJ structural and functional development remains unclear. In this study, mice with conditional inactivation of *neurofibromin 1* (*Nf1*) and *phosphatase and tensin homolog* (*Pten*), specifically in SCs, resulted in delayed NMJ maturation that led to delayed muscle growth, recapitulating the muscular dystrophy condition observed in human neurofibromatosis type I syndrome (NF1) patients. Expression levels of NMJ development related molecules such as *cholinergic receptor*, *nicotinic*, *alpha polypeptide 1* (*Chrna1*), *agrin* (*Agrn*), *dystrophin*, *muscular dystrophy* (*Dmd*), *laminin*, *beta 2* (*Lamb2*) and *dystroglycan 1* (*Dag1*) were also downregulated. To further explore the molecular alterations in these SCs, NF1- and PTEN-related pathways were analyzed in mutant sciatic nerves. As expected, hyperactive RAS/PI3K/AKT/mTOR signaling pathways were identified, suggesting the importance of these pathways for NMJ development, and subsequent muscle maturation.

The NMJ involves a chemical synapse containing three components: presynaptic motor neurons where acetylcholine is synthesized and released; postsynaptic muscle fibers where the acetylcholine receptors (AChR) are enriched; and PSCs covering the connection of motor neurons and muscle fibers (Barik *et al.* 2016). Previous studies have focused primarily on the cooperating actions of motor nerve terminals and muscle fibers, as well as the underlying molecular mechanisms that mediate synaptic activities. Recently, PSCs have been reported to play an important role in NMJ development and its maintenance (Darabid *et al.* 2014). For example, compromised NMJ formation and maturation can result in severe muscle diseases, such as myasthenia gravis and congenital myasthenia syndromes (Engel *et al.* 2003).

The formation, maturation and maintenance of reliable synapses at the NMJ require dynamic molecular interactions among the three previously mentioned components. Prior to innervation of the muscle fiber by the motor neuron, clusters of nerve-independent AChR pre-patterning occurs in muscle fibers at around embryonic day 12.5 (Lin *et al.* 2001). Muscle fibers of the NMJ are poly-innervated, with more than one axon input for each muscle fiber. Following this NMJ formation is its maturation, during which synapse elimination as well as rearrangement of both pre- and post-synaptic elements occurs (Stevens *et al.* 2007; Darabid *et al.* 2014). The extracellular proteoglycan agrin (AGRN) has been well documented to have a pivotal role in AChR clustering, mainly caused by the interaction of lipoprotein receptor-related protein 4 (LRP4) and muscle, skeletal, receptor tyrosine kinase (MUSK) complex (Kim *et al.* 2008; Zhang *et al.* 2008). Mutant mice lacking *Agrn* displayed pre-synaptic abnormalities such as aberrant axonal branching and arborization, and post-synaptic AChR disorganization (Gautam *et al.* 1996), whereas mutant mice lacking either *Lrp4* or *Musk* displayed severe and similar defects in AChR distribution (DeChiara *et al.* 1996; Weatherbee *et al.* 2006). Another extracellular molecule, neuregulin 1 (NRG1), promotes the biosynthesis of AChR proteins through binding of tyrosine kinase receptors from the epidermal growth factor receptor family (Ngo *et al.* 2012). In addition, WNT ligands are believed to be both positive and negative regulators of AChR aggregation and clustering via interactions with MUSK and AGRN (Budnik and Salinas 2011; Jing *et al.* 2009). Laminins are muscle-derived heterotrimeric glycoproteins and are major components in the NMJ basal lamina of the extracellular matrix. Mice lacking *Lamb2* displayed fewer synaptic vesicles and active zones for neurotransmitter exocytosis, whereas SCs in *Lamb2*-deficient mice were able to invade into the synaptic cleft (Knight *et al.* 2003). It is therefore clear that the formation and maintenance of NMJ requires dedicate coordination of specific molecular interactions.

The role of SCs in regulating NMJ development and maturation has been previously reviewed, providing comprehensive knowledge of the relationship between SCs and the maturation of pre- and post-synaptic elements at the NMJ (Darabid *et al.* 2014). Mice lacking *Nrg1*, *erb-b2 receptor tyrosine kinase 2* (*Erbb2*) or *erb-b2 receptor tyrosine kinase 3* (*Erbb3*) display an aberrant NMJ phenotype, which may have been caused by the absence of SCs in these animal models (Lin *et al.* 2000; Wolpowitz *et al.* 2000). Additionally, spatial ablation of SCs before and after innervation in mice also distorted NMJ development and its maintenance, further confirming the role of SCs in NMJ development (Barik *et al.* 2016). However, the molecular mechanism(s) underlying the functional roles of SCs in NMJ development and maturation remains to be elucidated.

*Nf1* and *Pten* are well-known tumor suppressor genes in different cancer types, and their co-mutations at specific tissues have been identified as the driver for tumor transformation (Keng *et al.* 2012; Chow *et al.* 2017). In our current study, mice with conditional inactivation of *Nf1* and *Pten*, specifically in SCs, displayed severe movement disorders. The status of NMJ development and maturation was investigated by characterizing markers of both pre-synaptic neurofilament (NF) and post-synaptic AChR. Our results indicated that *Nf1* and *Pten* inactivation in SCs delayed NMJ maturation at postnatal day 17 (P17). Expression of *Chrna1*, *Dmd*, *Agrn*, *Lamb2* and *Dag1* were all reduced in conditional *Nf1* and *Pten* inactivated mice (*desert hedgehog* (*Dhh*)*-Cre*; *Nf1*^flox/flox^; *Pten*^flox/flox^ or DNT mice), which further confirmed a dysfunctional NMJ maturation phenotype. To further explore the molecular changes in mutant SCs, protein expression of NF1- and PTEN-related molecules were analyzed using mutant mouse sciatic nerves. Hyperactive RAS, PI3K, AKT and mTOR were identified in the sciatic nerves of DNT mice revealing that NF1 and PTEN, as well as RAS/PI3K/AKT/mTOR pathways in SCs play essential roles in regulating NMJ maturation.

## Materials and Methods

### Breeding strategy of DNT mice

**Figure 1:**
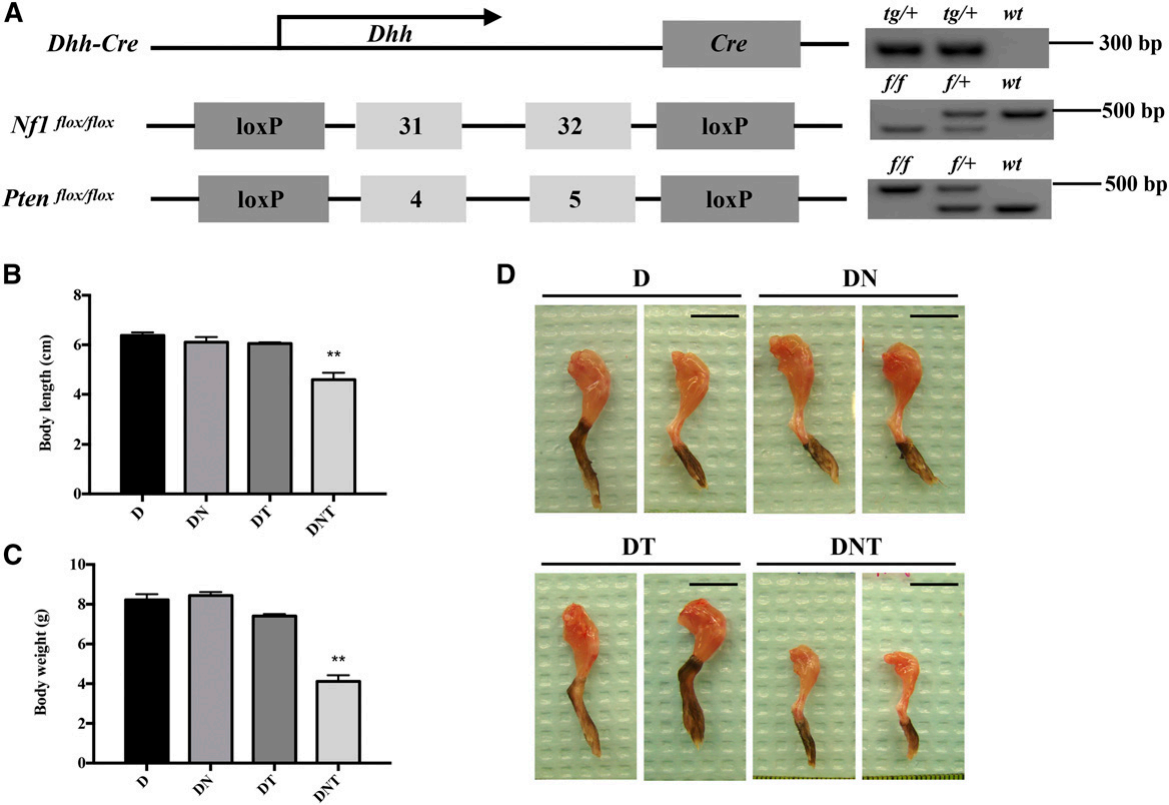
Conditional *Nf1* and *Pten* inactivation in SCs resulted in reduced musculature in DNT mice. (A) Genetic profile of Cre-loxP system in DNT mice, and representative PCR bands for the genotyping result of *Dhh*, *Nf1* and *Pten*. *tg*, transgene; *f*, floxed allele; *wt*, wild-type allele. (B) Body length of all P17 mouse experimental cohorts: D, DN, DT and DNT. (C) Body weight of all P17 mouse experimental cohorts: D, DN, DT and DNT. Data expressed as mean ± SEM; **P < 0.01 (unpaired Student’s *t*-test); *n* = 7 mice per group. (D) Representative pictures of hind limbs from P17 D, DN, DT and DNT cohorts. Scale bars, 1cm.

Detailed breeding strategy of experimental mice cohorts can be found in our previous study (Keng *et al.* 2012). Primers used for genotyping of transgenes were performed as previously described (Keng *et al.* 2012). PCR bands representing different genotyping results of transgene combinations as shown in Figure 1A. All animal work was done under the regulation and guidelines of Centralized Animal Facility (CAF) and Animal Subjects Ethics Review (ASESC), The Hong Kong Polytechnic University, as well as The Laboratory Animal Services Centre (LASEC), The Chinese University of Hong Kong.

### Immunofluorescence staining of NMJ

Both hind limbs were dissected from P17 control and DNT mice following euthanasia by carbon dioxide. These harvested fresh limbs were fixed in 10% formalin (Sigma-Aldrich, Missouri, USA) with rotation overnight. Fixed limbs were rinsed three times for 30 min with phosphate buffered saline (PBS) (Life Technologies, California, USA) at room temperature. The tibialis anterior (TA) muscle was isolated from the fixed limbs and further teased into smaller muscle fibers under the dissecting microscope. Samples were placed in a 24-well plate and incubated in 0.1 M glycine diluted with PBS for one hour. To increase permeability, samples were rinsed three times with 0.5% Triton X-100 in PBS, 30 min for each rinse. The samples were then treated with blocking buffer (2.5% bovine serum albumin in 0.5% Triton X100/PBS) and incubated at either 4 hr at room temperature or overnight at 4°. Antibodies used for immunofluorescent microscopy were: neurofilament (NF), Alexa Fluor 555 Conjugate (Sigma-Aldrich) and AChR labeled using α-Bungarotoxin (α-BTX), Alexa Fluor 488 conjugate (Thermo Fisher Scientific, Massachusetts, USA). NF (1:500) and α-BTX (1:250) were added to samples and incubated for two days at 4°, then washed by rinsing three times, each for 1 hr, with 0.5% Triton X100/PBS. Muscle fiber samples were then placed on glass slides, followed by mounting with ProLong Antifade Kit (Thermo Fisher Scientific).

### Quantitative RT-PCR

Muscle tissue from the hind limbs was homogenized in TRIzol reagent (Invitrogen, California, USA), using the Bertin Technologies Precellys Evolution Homogenizer. RNA was extracted following the manufacturer’s instructions. Complementary DNA (cDNA) was synthesized by using the PrimeScript RT Master Mix (TaKaRa Bio Inc., Kusatsu, Japan). Quantitative RT-PCR was conducted using GoTaq qPCR Master Mix (Promega, Wisconsin, USA). *Ribosomal protein S20 (Rps20)* was used as a reference gene to normalize the mRNA expression of *Chrna1*, *Dmd*, *Agrn*, *Lamb2* and *Dag1*. The following primer pairs were used: *Rps20*-For, 5′- TGCTGAGGAACAAGTCGGTC-3′; *Rps20*-Rev, 5′- AGTCCGCACAAACCTTCTCC-3′; *Chrna1*-For, 5′-TATAACAACGCAGACGGCGA-3′; *Chrna1*-Rev, 5′-GCTGGTCACTTTCCGGGTTA-3′; *Dmd*-For, 5′- CTCACTGCCTGTGAAACCCT-3′; *Dmd*-Rev, 5′- CAGGCTCAAGAGATCCAAGCA-3′; *Agrn*-For, 5′- CTAGGGGAATCTCCGGTCCC-3′; *Agrn*-Rev, 5′- CCCATTAAGGCAGGGGTTGT-3′; *Lamb2*-For, 5′- ACCCACACGGTCGGGATG-3′; *Lamb2*-Rev, 5′- ACAGCCAGGTACATCCAAGG-3′; *Dag1*-For, 5′- GAGGGACTGGAAGAACCAGC-3′; *Dag1*-Rev, 5′- CCTGCTGCAGACACCTTGAT-3′.

### Western blot analysis

Protein was extracted from sciatic nerves of experimental animals using the Qproteome Mammalian Protein Prep Kit (Qiagen, Hilden, Germany) following the manufacturer’s instructions. Protein concentrations were determined using the Bradford Protein Assay (Bio-Rad, California, USA). Protein samples were separated either on an 8% or 12% SDS-PAGE gel, before transferring to polyvinylidene difluoride (PVDF) membranes (MilliporeSigma, Massachusetts, USA). PVDF membranes were first incubated overnight with primary antibodies at 4°. Antibodies purchased from Cell Signaling Technology (Massachusetts, USA) and the dilutions used were as follows: AKT (1:2000), phospho-AKT (1:2000), PI3K (1:2000), phospho-PI3K (1:2000), phospho-mTOR (1:2000), RAS (1:2000) and ACTB (1:2000). This was followed by incubation with the corresponding secondary antibody at room temperature for one hour. Targeted bands were detected using a horseradish peroxidase-conjugated chemiluminescent kit (MilliporeSigma). ACTB was used as a loading control.

### Data availability

The authors confirm that all data necessary for supporting the conclusions of the article are present within the article.

## Results

### Conditional inactivation of Nf1 and Pten in SCs resulted in reduced musculature

The DNT (*Dhh-Cre*; *Nf1*^flox/flox^; *Pten*^flox/flox^) mice have been established as an animal model of malignant peripheral nerve sheath tumor in our previous study (Keng *et al.* 2012). In addition, DNT mice were smaller in size compared with control mice D (*Dhh-Cre*), single gene mutant mice DN (*Dhh-Cre*; *Nf1*^flox/flox^) and DT (*Dhh-Cre*; *Pten*^flox/flox^) at postnatal day 7 (P7). However, there were no observable size differences between D and DT/DN mice. Most importantly, DNT mice have a tremoring phenotype, displayed difficulty in standing and obvious movement disability. This observation implies that DNT mice may have a deficit in muscle development and/or its maturation. DNT mice were killed at P17 before they became moribund and their body measurements taken (body length and weight). DNT mice were significantly shorter than control mice D, with an average body length of 4.6 cm and 6.4 cm, respectively (Figure 1B), while the body lengths of DN and DT mice had no significant differences compared with that of control mice D (Figure 1B). DNT mice had an average body weight of 4.1 g, while control D mice were on average 8.2 g (Figure 1C). The musculature of the DNT mice, including the limb muscles, diaphragm and heart were macroscopically reduced compared to control mice D (*data not shown*). The representative images of hind limb muscles taken from experimental and control cohorts are shown in Figure 1D.

### Conditional inactivation of Nf1 and Pten in SCs induced NMJ maturation deficits

**Figure 2:**
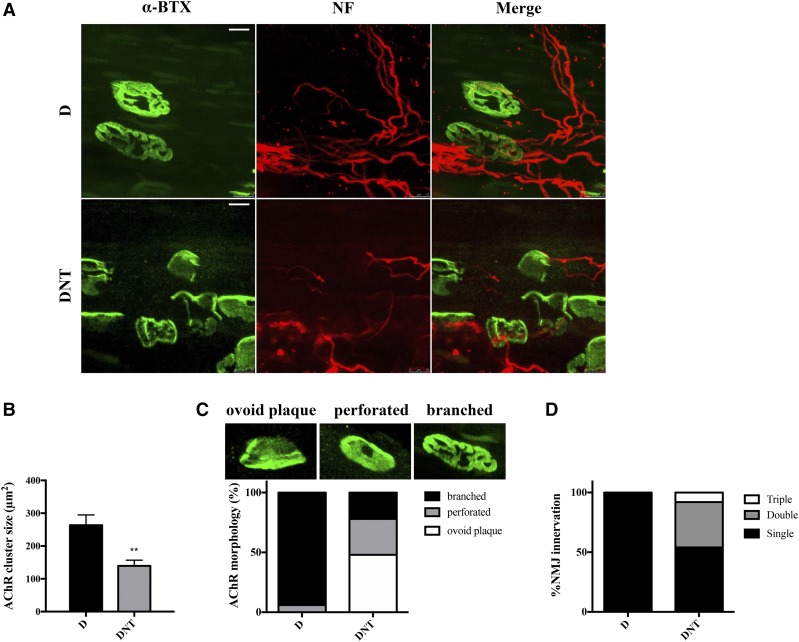
NMJ maturation deficits in TA muscle of DNT mice. (A) Representative confocal images of TA muscles from P17 D and DNT mice. Teased TA muscles were stained with NF conjugate Alexa Fluor 555 to label pre-synaptic axon, and α-BTX conjugate Alexa Fluor 488 for visualization of AChR clustering. Scale bars, 10 µm. (B to D) Quantitative analyses of the AChR cluster size (B), AChR morphology (C) and NMJ innervation (D). Data expressed as mean ± SEM; **P < 0.01 (unpaired Student’s *t*-test).

In order to investigate the underlying mechanism of the movement phenotype in DNT mice, the NMJ structure in mouse TA muscle was explored. NF conjugate Alexa Fluor 555 was used to label pre-synaptic axons, and α-BTX conjugate Alexa Fluor 488 was used to label the post-synaptic AChRs. NMJ morphology of P17 DNT mice revealed distorted AChR clusters and delayed synapse elimination, indicating a delay in NMJ synapse maturation (Figure 2A). In DNT mice, the average AChR endplate was 139.6 µm^2^ in size, compared with 263.6 µm^2^ for control mice D (Figure 2B). AChR-enriched postsynaptic elements are ovoid plaque shaped at P1, followed by perforation with a lower density of AChR clusters and branched AChR clusters during the first two weeks of early postnatal age (Sanes and Lichtman 2001; Marques *et al.* 2000). Therefore, the transition of ovoid plaque to branched pretzel-shape of AChR clusters is indicative of the NMJ maturation process. The percentage of branched AChR clusters in DNT mice were dramatically reduced compared to that in control mice D (Figure 2C). Instead, increased plaque shaped and perforated endplates in DNT mice were observed (Figure 2C), indicating a delayed endplate maturation phenotype. In addition, the percentage of poly-innervated AChRs increased, indicating a delayed synapse elimination phenotype (Figure 2D). Taken together, these results imply that NMJ maturation was delayed in DNT mice.

### Molecular confirmation of NMJ maturation deficits in the TA muscle of DNT mice

**Figure 3:**
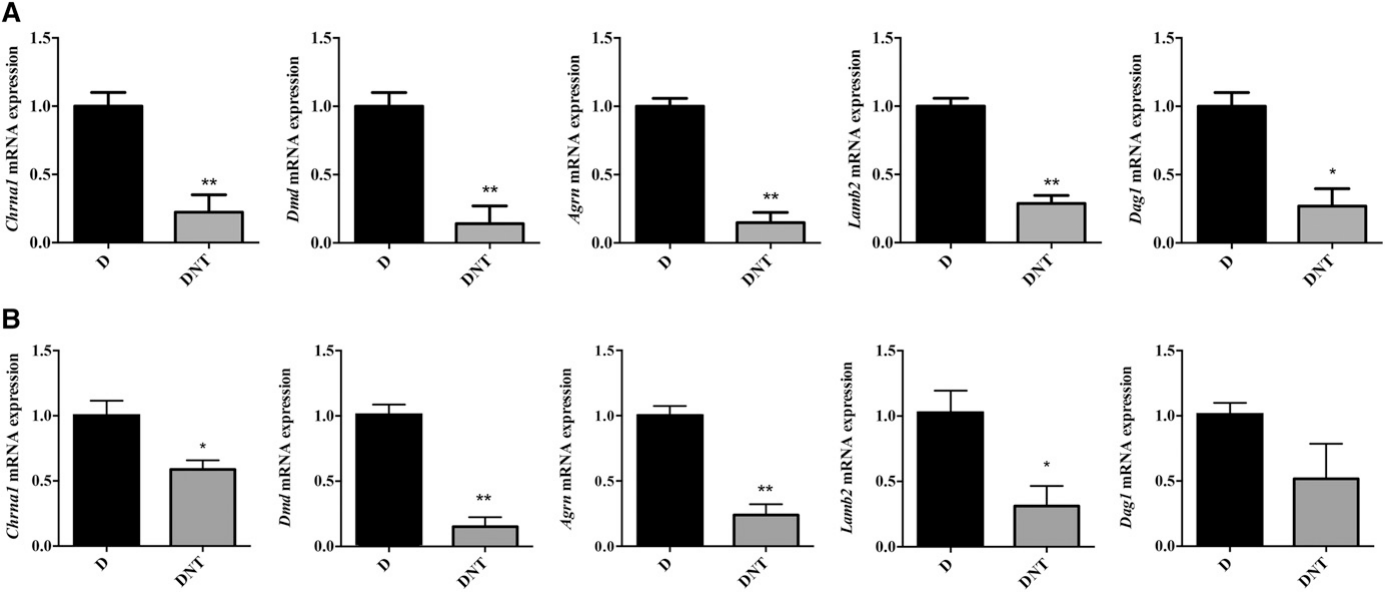
Molecular confirmation of NMJ maturation deficits in DNT mice. Expression levels of *Chrna1*, *Dmd*, *Agrn*, *Lamb2* and *Dag1* were reduced in TA muscle (A) and diaphragm (B) of DNT mice by quantitative real-time PCR. Data expressed as mean ± SEM of three independent experiments. *P < 0.05 and **P < 0.01 (unpaired Student’s *t*-test).

To further study the effects of *Nf1* and *Pten* inactivation on NMJ growth, *Chrna1*, *Dmd*, *Agrn*, *Lamb2* and *Dag1* were analyzed using RNA extracted from both TA muscle and diaphragm. *Chrna1* gene (also known as *Achr-1*) encodes for an alpha subunit of the muscle-derived nicotinic acetylcholine receptor, which also serves as a marker for post-synaptic muscle fiber. DMD connects transmembrane components of the dystrophin-glycoprotein complex to the intracellular cytoskeleton network (Gao and McNally 2011). Mice lacking *Dmd* displayed NMJ malformation and subsequent muscle dystrophy (Shiao *et al.* 2004). *Agrn* and synaptic basal lamina, especially *Lamb2*, are essential for pre- and post-synaptic maturation and maintenance of NMJ (Nishimune *et al.* 2008; Gautam *et al.* 1996). DAG1 works as a transmembrane protein, linking it to the ECM (Smith *et al.* 2013). DAG1 also acts as a receptor for laminin and AGRN in the regulation of nerve-induced AChR clustering formation (Tremblay and Carbonetto 2006). In our study, mRNA expression of *Chrna1* was reduced in DNT mice (Figure 3A and 3B), consistent with the smaller endplate size (Figure 2B). The expression of *Dmd*, *Agrn*, *Lamb2* and *Dag1* in TA muscle (Figure 3A) and diaphragm (Figure 3B) were reduced in DNT mice compared with that in control mice D. Taken together, these results strongly suggest dysfunctional NMJ maturation in DNT mice.

### Activated RAS/PI3K/AKT/mTOR pathways in sciatic nerves of DNT mice

**Figure 4:**
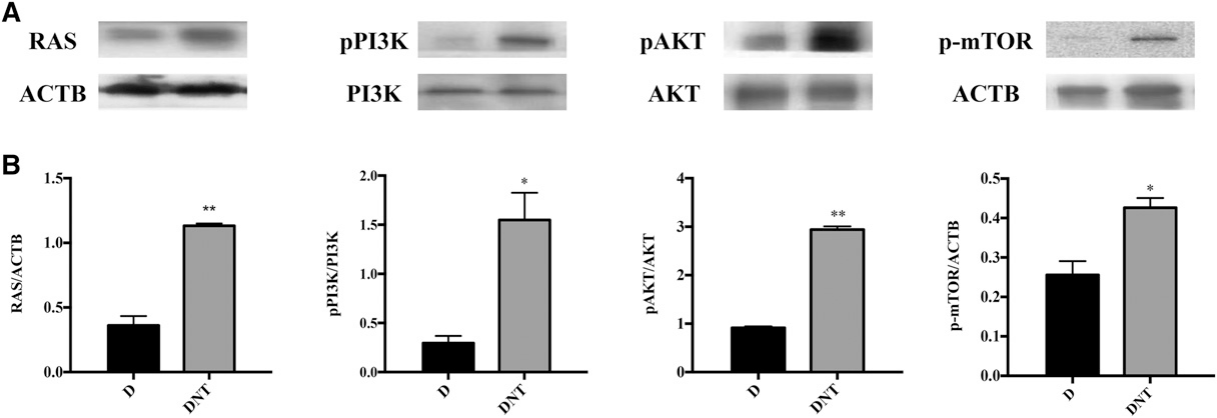
Activation of RAS/PI3K/AKT/mTOR pathways in sciatic nerves of DNT mice. (A) Representative Western blots showing significantly higher levels of RAS, phosphorylated PI3K (pPI3K), phosphorylated AKT (pAKT) and phosphorylated mTOR (p-mTOR) in DNT compared with control D cohorts. (B) Semi-quantitative analyses of Western blots. RAS and p-mTOR levels relative to ACTB; while pPI3K and pAKT levels relative to total PI3K and total AKT levels, respectively. Data expressed as mean ± SEM of three independent blots. *P < 0.05 and **P < 0.01 (unpaired Student’s *t*-test).

In order to elucidate the molecular mechanism(s) caused by *Nf1* and *Pten* inactivation in SCs, protein expression was evaluated using the protein lysate taken from sciatic nerves of mutant DNT mice. It has been reported that NF1 is a negative regulator of RAS and its mutation induces RAS activation, as well as downstream PI3K signaling pathway (Bollag *et al.* 1996). As expected, RAS and PI3K phosphorylation were increased in DNT mice (Figure 4A and 4B). PTEN works as a negative mediator of AKT, therefore its mutation results in activated AKT phosphorylation (Stambolic *et al.* 1998). AKT phosphorylation and its downstream mTOR signaling pathway were significantly upregulated in DNT mice compared with that of control mice D (Figure 4A and 4B). These results suggest that *Nf1* and *Pten* inactivation in SCs induce hyperactive RAS/PI3K/AKT/mTOR pathways, affecting NMJ maturation in DNT mice.

## Discussion

In this study, characterization and molecular alterations underlying the NMJ abnormalities were evaluated in mice with conditional inactivation of *Nf1* and *Pten* genes, specifically in SCs. In addition, NF1 and PTEN related RAS/PI3K/AKT/mTOR pathways in SCs were proven to have a pivotal role in regulating NMJ maturation.

DNT mice have a short lifespan of around P18-20 days, as these mice were reported to bear high grade malignant peripheral nerve sheath tumors (Keng *et al.* 2012). Importantly, the tremoring, difficulty in movement and runty phenotype can be observed as early as P7. Comparing with control mice (D, DN and DT), the heart and diaphragm from DNT mice were significantly smaller at P17 (data not shown), indicating these mice may also has a severe muscle developmental disorder, contributing to its short lifespan. Clinically, patients with NF1 are reported to have reduced skeletal muscle size, muscle weakness and motor function deficits due to nerve dysfunction (Summers *et al.* 2015). These symptoms are highly consistent with the muscle phenotype of DNT mice but the detailed mechanism(s) underlying the movement disorders are largely unknown. Recently, SCs have drawn much attention regarding their role in regulating NMJ development (Darabid *et al.* 2014; Barik *et al.* 2016). Based on these previous studies, we hypothesized that conditional inactivation of *Nf1* and *Pten* in SCs could also affect NMJ development and subsequent muscle growth/maturation.

To further explore this, the body length and weight of all experimental mice were examined. The body length and weight of DNT mice were significantly reduced compared with either control mice D or single gene mutated mice DN/DT (Figure 1B and 1C). A reduction in musculature was observed in DNT mice but not in DN or DT mice, indicating synergistic effects of *Nf1*- and *Pten*-deficiency in SCs contributing to muscle dystrophy (Figure 1D). Since SCs have been shown to be involved in AChR cluster maturation and maintenance (Barik *et al.* 2016), the NMJ structure in DNT mouse TA muscle was also investigated. Compared with control mice D, the NMJ phenotype in DNT mice included an AChR endplate size reduction and the percentage of branched endplate and mature single innervated endplate were decreased (Figure 2). In addition, molecules known to be key markers and mediators in NMJ development such as *Chrna1*, *Dmd*, *Agrn*, *Lamb2* and *Dag1* (Nishimune *et al.* 2008; Jacobson *et al.* 2001; Davies and Nowak 2006) were also decreased in DNT mice compared with that of control group D (Figure 3). Importantly, SCs have been shown to be a source for AGRN, WNT ligands, NRG1 and TGFB (Darabid *et al.* 2014). Dysfunctional SCs could therefore account for the abnormal NMJ maturation observed in DNT mice.

However, it remains unclear which different roles the myelinating SCs and PSCs (non-myelinating Schwann cells) may play in the NMJ maturation process. It has been shown that targeting PSCs in mice using anti-disialoside antibodies from patients have resulted in both morphological and functional changes in NMJ (Halstead *et al.* 2004). In addition, PSCs are believed to participate in NMJ remodeling after nerve injury by guiding the reinnervation of axons (Kang *et al.* 2014). In addition, the AKT/mTOR pathway has been shown to regulate myelination development in the peripheral nervous system (Domènech-Estévez *et al.* 2016; Norrmén and Suter 2013). PTEN reduction in both oligodendrocytes and SCs induced hypermyelination while its activation resulted in blocked myelination (Goebbels *et al.* 2010). Other molecules such as phosphatidylinositol 4-kinase alpha and fatty acid synthase have been reported to cause aberrant myelination (Alvarez-Prats *et al.* 2018; Montani *et al.* 2018), which suggests that the AKT/mTOR pathway can affect the myelination of SCs and influence the actions of neurons. Therefore, the exact roles of myelinating SCs and PSCs in NMJ development remains unclear. Further studies should be conducted in order to elucidate the regulatory effects of specific SCs in NMJ development and maturation. It has also been demonstrated the ablation of SCs can influence NMJ maturation and its maintenance, but the exact mechanism involved with this also remains elusive (Barik *et al.* 2016).

Finally, our current study demonstrates that hyperactive RAS/PI3K/AKT/mTOR pathways in SCs could disrupt expression of basal laminin molecules including *Agrn*, *lamb2*, *Dag1*, *Chrna1* and *Dmd*, resulting in delayed NMJ maturation. This study therefore provides additional genetic understanding to the role of SCs in NMJ development and maturation.
